# Network of ictal head version in mesial temporal lobe epilepsy

**DOI:** 10.1002/brb3.1820

**Published:** 2020-08-28

**Authors:** Yao Wang, Xiu Wang, Lin Sang, Chao Zhang, Bao‐tian Zhao, Jia‐jie Mo, Wen‐han Hu, Xiao‐qiu Shao, Feng Wang, Lin Ai, Jian‐guo Zhang, Kai Zhang

**Affiliations:** ^1^ Department of Neurosurgery Beijing Tiantan Hospital Capital Medical University Beijing China; ^2^ Beijing Key Laboratory of Neurostimulation Beijing China; ^3^ Epilepsy Center Peking University First Hospital Fengtai Hospital Beijing China; ^4^ Stereotactic and Functional Neurosurgery Laboratory Beijing Neurosurgical Institute Capital Medical University Beijing China; ^5^ Department of Neurology Beijing Tiantan Hospital Capital Medical University Beijing China; ^6^ Department of Neurosurgery General Hospital of Ningxia Medical University Ningxia China; ^7^ Department of Nuclear Medicine Beijing Tiantan Hospital Capital Medical University Beijing China

**Keywords:** epileptogenicity index, head version, network, positron emission tomography, temporal lobe epilepsy

## Abstract

**Objective:**

Ictal head version is a common clinical manifestation of mesial temporal lobe epilepsy (MTLE). Nevertheless, the location of the symptomatogenic zone and the network involved in head version remains unclear. We attempt to explain these problems by analyzing interictal ^18^FDG‐PET imaging and ictal stereo‐electroencephalography (SEEG) recordings in MTLE patients.

**Methods:**

Fifty‐eight patients with MTLE were retrospectively analyzed. The patients were divided into version (+) and (−) groups according to the occurrence of versive head movements. The interictal PET data were compared among 18 healthy controls and the (+) and (−) groups. Furthermore, epileptogenicity index (EI) values and correlations with the onset time of head version were analyzed with SEEG.

**Results:**

Intergroup comparisons showed that PET differences were observed in the middle temporal neocortex (MTN), posterior temporal neocortex (PTN), supramarginal gyrus (SMG), and inferior parietal lobe (IPL). The EI values in the SMG, MTN, and PTN were significantly higher in the version (+) group than in the version (−) group. A linear relationship was observed between head version onset and ipsilateral onset time in the SMG, orbitofrontal cortex (OFC), MTN, and PTN. A linear relationship was observed between EI, the difference between version onset and temporal neocortex onset, and the y‐axis of the MNI coordinate.

**Conclusion:**

The generation of ictal head version contributes to the propagation of ictal discharges to the intraparietal sulcus (IPS) area. The network of version originates from a mesial temporal lobe structure, passes through the MTN, PTN, and SMG, and likely ends at the IPS.

## Graphical abstract

This is a research of semiology of mesial temporal lobe epilepsy. It mainly demonstrated the network of the version of head and eyes.

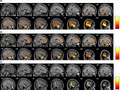

## 1 INTRODUCTION

Head version is a common seizure phenomenon in patients with focal and primary generalized seizures (Chin & Miller, 2004; Rheims et al., 2005; Wyllie, Luders, Morris, Lesser, & Dinner, 1986). It is defined as involuntary and forced head movements consisting of at least 45° of rotation or tilt (Marks & Laxer, 1998). Constantly accompanied with versive eye movements (Baysal‐Kirac et al., 2015; Marks & Laxer, 1998), head version can be observed in frontal (Rheims et al., 2005), temporal (Marks & Laxer, 1998), and parietal (Bartolomei et al., 2011; Salanova, Andermann, Rasmussen, Olivier, & Quesney, 1995) lobe epilepsy. It occurs more often in temporal and parietal lobe epilepsy. In temporal lobe epilepsy, head version occurs shortly before generalization in 27% of patients and during focal seizures in 45% of patients (Marks & Laxer, 1998). These proportions are higher in parietal lobe epilepsy (PLE), in which ictal head version was observed in 65% of patients (Bartolomei et al., 2011).

However, the generation of ictal head version and the involved network is still unclear. Previous studies have suggested that the head turning observed in frontal lobe epilepsy (FLE) is due to the spread of ictal discharges to the frontal eye fields (FEF) (Godoy, Luders, Dinner, Morris, & Wyllie, 1990; Remi et al., 2011). The mechanism of head turning in temporal lobe epilepsy (TLE) is likely similar to that observed in FLE (Shin, Hong, Tae, & Kim, 2002). However, this inference has not been confirmed. In TLE, the onset time of head turning is later than that observed in FLE, suggesting that the involved network may be more complex (Remi et al., 2011). In both macaque and human studies, several eye fields and visual‐motor pathways have been identified (Papadopoulos, Sforazzini, Egan, & Jamadar, 2018; Peuskens, Sunaert, Dupont, Van Hecke, & Orban, 2001; Saito et al., 1986). At present, medial superior temporal area (MST) and intraparietal sulcus (IPS) regions have been demonstrated to be related to visual motion and may also be involved in controlling the movement of the head and eyes (Peuskens et al., 2001; Saito et al., 1986).

To determine the mechanism of head version in TLE and identify potentially involved brain regions, we analyzed the anatomo‐electrical characteristics of head version in patients with medically refractory MTLE who underwent anterior temporal lobectomy (ATL) and were seizure‐free.

## 2 MATERIALS AND METHODS

### 2.1 Patient selection

Fifty‐eight consecutive patients with MTLE were retrospectively recruited between January 2015 and January 2018. A total of 186 seizures captured on video electroencephalography (EEG) were reviewed. Patients with involuntary and forced head movements consisting of at least 45° of rotation or tilt occurring at the beginning of or during seizures were allocated to the version (+) group. Others were placed in the version (−) group. The semiology and groups were confirmed by two senior epileptologists. For the few divergent cases, the decision was made by group discussion. To localize the seizure onset zone (SOZ), all patients underwent evaluation by semiology, EEG, MRI, and [^18^F]‐fluorodeoxyglucose positron emission tomography (^18^FDG‐PET). Stereo‐electroencephalography (SEEG) was performed when the SOZ remained uncertain based on the previous evaluation. After preoperative evaluation, all the SOZs were located in the unilateral hippocampus. All patients underwent ATL and were seizure‐free for at least 12 months of follow‐up. Eighteen healthy volunteers were included in this study (male: *n* = 10, age: 22.6 ± 3.3 years old). These volunteers were free of neurological or psychiatric disorders, and their MRI scans were normal and could be considered representative of the normal population. The study, including informed consent and protocols, was approved by the Institutional Review Boards (IRB) of Beijing Tiantan Hospital, Capital Medical University.

### 2.2 18FDG‐PET data acquisition and analysis

All patients and the 18 healthy controls underwent interictal PET scans with the same protocols. No patients had clinical seizures less than 6 hr before or during the PET scan. The PET examinations were performed under standard resting conditions using a GE Discovery ST PET‐CT system (300 mm FOV, matrix 192 × 192, 3.27 mm slice thickness). Whole‐brain statistical analysis was performed at the voxel level using SPM8 software (Wellcome Department of Cognitive Neurology, University College, London, UK). Whole‐brain statistical analysis was performed at the voxel level using SPM8 software (Wellcome Department of Cognitive Neurology, University College, London, UK). The PET images of patients with right SOZ were transposed horizontally to the left. Then, the images were spatially normalized (voxel size: 2 mm × 2 mm × 2 mm) and smoothed with a Gaussian filter (8 mm FWHM). The images obtained in the version (+) and version (−) groups were compared with those obtained in a group of 18 healthy controls with the age and gender as covariates using voxel‐based independent *t* test analysis, as implemented in SPM8 software. Similarly, the version (+) and (−) groups were compared with the existence of focal to bilateral seizures as a covariate. In the version (+) group, the comparison was performed in patients with ipsilateral and contralateral version. The brain regions with differences in comparison between groups were selected as ROIs. Then, SEEG was used to confirm whether the selected ROIs were involved in the epileptic network. The definition of brain area was based on the anatomical automatic labeling (AAL) atlas. The SPM maps were thresholded using *p* < .001 corrected for multiple comparisons with the false discovery rate method (FDR‐corrected). The cluster threshold was set to 100 voxels.

### 2.3 SEEG recording

Long‐term recordings were acquired using intracerebral multiple contact electrodes (8–16 contacts, length: 2 mm, diameter: 0.8 mm, 1.5 mm apart, Beijing HKHS Healthcare Co., Ltd). In patients who underwent SEEG, several seizures need to be captured. Signals were recorded on a NIHON KOHDEN video‐EEG monitoring system with a sampling rate of 1,000 Hz or 2,000 Hz. Postoperative computerized tomography (CT) was performed routinely. The accuracy of electrode implantation positions was determined by MRI/CT coregistration.

All ictal SEEG signals were visually reviewed by two senior epileptologists, who discussed the results together. The SOZ of each seizure recorded by SEEG was first interpreted manually. Moreover, visual analyses allowed us to identify the brain regions involved in each seizure. When the ictal discharge spread to the regions of interest (ROIs), a change from background was observed. We recorded the earliest time that the SEEG changed from background activity as the onset time of the ROI (Aupy et al., 2018).

### 2.4 Ictal SEEG processing

After visual analysis, the epileptogenicity index (EI) was calculated for all selected seizures using a plug‐in implemented in Anywave software (available at http://meg.univ‐amu.fr/wiki/AnyWave). As described in a previous study (Bartolomei, Chauvel, & Wendling, 2008), EI is a semiautomatic quantified method used to compute the SEEG signal to identify the SOZ and propagation zones (Lagarde et al., 2018). In general, the EI index is determined by two conditions. The energy ratios of higher frequency bands (beta, gamma, high gamma, and ripple) and lower frequency bands (theta, alpha) and the occurrence time of low‐voltage fast activity (LVFA) were recorded. Finally, the EI was normalized and ranged from 0 (no epileptogenicity) to 1 (maximal epileptogenicity). An EI value of 1 was considered to indicate the onset of seizures. Results that were the same as those obtained in the manual interpretation were retained for subsequent analysis.

For each patient, we defined regions of interest (ROIs) according to the anatomical automatic labeling (AAL) atlas. The major ROI SEEG electrodes passed through the amygdala, parahippocampal gyrus, anterior insula, posterior insular, frontal operculum, rolandic operculum, supramarginal gyrus (SMG), posterior cingulate cortex (PCC), orbitofrontal cortex (OFC), middle frontal gyrus, and temporal neocortex. Coregistration images obtained on MRI/CT after SEEG were standardized to Montreal Neurological Institute (MNI) space. Based on the y‐axis of the MNI coordinates, the temporal lobe neocortex was roughly divided into three parts: anterior temporal neocortex (ATN) (MNI coordinate >−20), middle temporal neocortex (MTN) (−20 ≤ MNI coordinate <−40), and posterior temporal neocortex (PTN) (MNI coordinate ≤−40).

### 2.5 Statistical analysis

Descriptive statistics were applied to analyze the distributions of clinical data. Data with a normal distribution are expressed as the mean ± *SD*, while those with non‐normal distributions are expressed as the median and quartile. *T* tests and one‐way ANOVA were applied to the analysis of measurement data with a normal distribution. The Mann–Whitney test was used to analyze intergroup EI values because of the non‐normal distribution in each group. Linear regression was used to calculate the correlation between the onset times of different brain regions and head version. The same method was used to analyze the correlation between the MNI coordinates in the temporal neocortex and the differences in onset time from head version to the temporal neocortex. All data were analyzed using SPSS software (SPSS, version 24.0 for Windows; SPSS Inc.). *p*‐values <.05 were considered significant.

## 3 RESULTS

### 3.1 Patient characteristics

**Figure 1:**
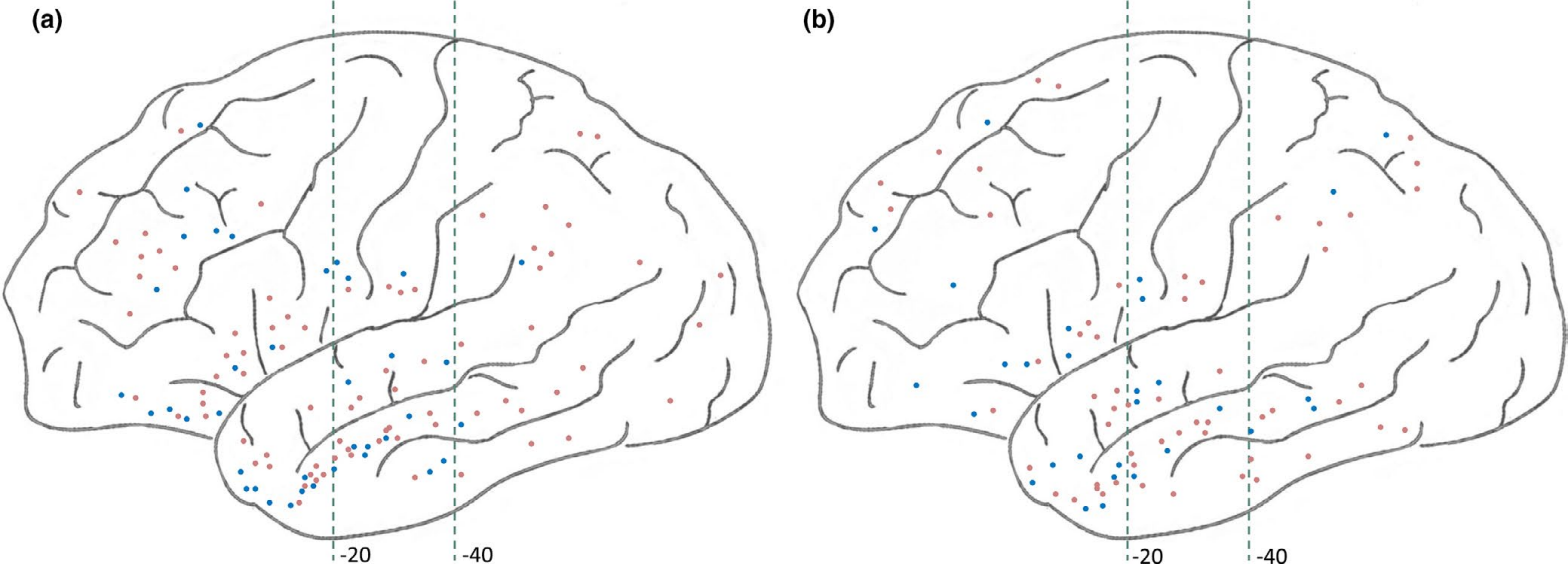
Lateral view of SEEG implantation in the version (+) (a) and (−) (b) groups. The blue dots correspond to the right‐sided electrodes, and the orange dots correspond to the left‐sided electrodes. The green dashed line represents the y‐axis of the MNI coordinates at −20 and −40

A total of 58 patients (male, *n* = 27) were included in the analysis and had a median/mean age of seizure onset of 13.4 years old (range: 0.5–28 years old), surgery age of 26.10 ± 7.09 years old (range: 10–39 years old), and epilepsy duration of 12.44 ± 7.72 years (range: 1–33 years). Twenty‐one patients were classified as version (+), while 37 patients were version (−). There was no significant difference in age and gender between the version (+) and (−) groups compared with the 18 healthy volunteers. In the version (+) group, 13 patients demonstrated ipsilateral head version to the resective side. The demographic and clinical features of the two groups are shown in Table 1. In all, focal to bilateral seizures were observed in 7 version (+) and 5 version (−) patients (*p* = .097). Other semiologies between groups showed no statistical difference. SEEG electrodes were implanted in 22 patients after discussion (these implantation plans are shown in Figure 1). The detailed clinical information of the 22 patients is shown in Table 2. Hippocampal sclerosis was the only visible abnormality evaluated by preoperative MRI. Based on noninvasive and SEEG evaluation, the hippocampus was considered the SOZ in all patients. All patients underwent ATL and were seizure‐free after an average of 19.76 ± 6.64 months (range: 12–35 months) of follow‐up. Hippocampal sclerosis was confirmed by pathology in all patients.

**Table 1:** Demographic and clinical features of the 58 patients

	version (+)	version (−)	Intergroup statistics
Sex (M/F)	13/8	14/23	*X* ^2^ = 3.119 *p* = .077
Side (L/R)	11/10	23/14	*X* ^2^ = 0.528 *p* = .467
Frequency (*n*/month median)	4	5	*Z* = −1.311 *p* = .190
Onset age (years)	10.72 ± 6.54	15.32 ± 7.06	*t* = −2.448 *p* = .018
Surgery age (years)	25.33 ± 6.74	26.44 ± 7.34	*t* = −0.620 *p* = .538
Duration (years)	14.61 ± 7.90	11.22 ± 7.45	*t* = 1.631 *p* = .109

**Table 2:** Patient and epilepsy characteristics for implants placed in SEEG

	Sex	Onset age	duration	Operative side	Auras	Version side	Associated signs
1	F	7	5	Left	Uncomfortable feeling in the heart	To left	Behavioral arrest, focal to bilateral seizure
2	F	9	28	Left	Palpitation	To left	Blinking
3	F	12	14	Left	Undefined	To left	Swallowing
4	M	6	19	Right	fear	To right	Behavioral arrest, left‐hand automatisms
5	M	1	28	Left	NA	To left	Behavioral arrest, blinking, left‐hand automatisms
6	F	22	11	Right	Déjà vu	To left	Upper limbs tonic
7	M	5	16	Left	Headache	To left	Left‐hand automatisms
8	F	8	19	Left	Rising epigastric sensation	To right	Hand automatisms, swallowing, focal to bilateral seizure
9	F	12	12	Right	Fear	To left	Hand automatisms
10	M	8	11	Right	Déjà vu	To right	Left upper limb flexion
11	M	28	11	Left	NA	To left	Blinking, pouting
12	F	8	14	Right	NA	To left	Shouting, right‐hand flapping
13	M	13	3	Right	Nausea	To right	Behavioral arrest, head version to right, lip smacking, focal to bilateral seizure
14	M	6	19	Left	Palpitation	To left,	Behavioral arrest, left‐hand automatisms
15	F	24	2	Right	Nausea	NA	Left‐hand automatisms, peddling, chewing
16	M	3	23	Left	Bellyache	NA	Hand automatisms, chewing
17	M	8	3	Left	Uncomfortable feeling in the heart	NA	Behavioral arrest
18	M	13	16	Left	NA	NA	Upper limb tonic, paddling, hand automatisms, focal to bilateral seizure
19	F	13	6	Right	Rising epigastric sensation	NA	Swallowing, left‐hand dystonia, right‐hand automatisms
20	F	14	2	Left	Déjà vu	NA	Left‐hand automatisms, lifting, focal to bilateral seizure
21	M	12	10	Left	Undefined	NA	Right‐hand lifting
22	F	7	15	Right	Fear	NA	Behavioral arrest, head and eyes turn to left, lip smacking, right‐hand automatisms

Abbreviation: NA, not applicable.

### 3.2 PET findings

**Figure 2:**
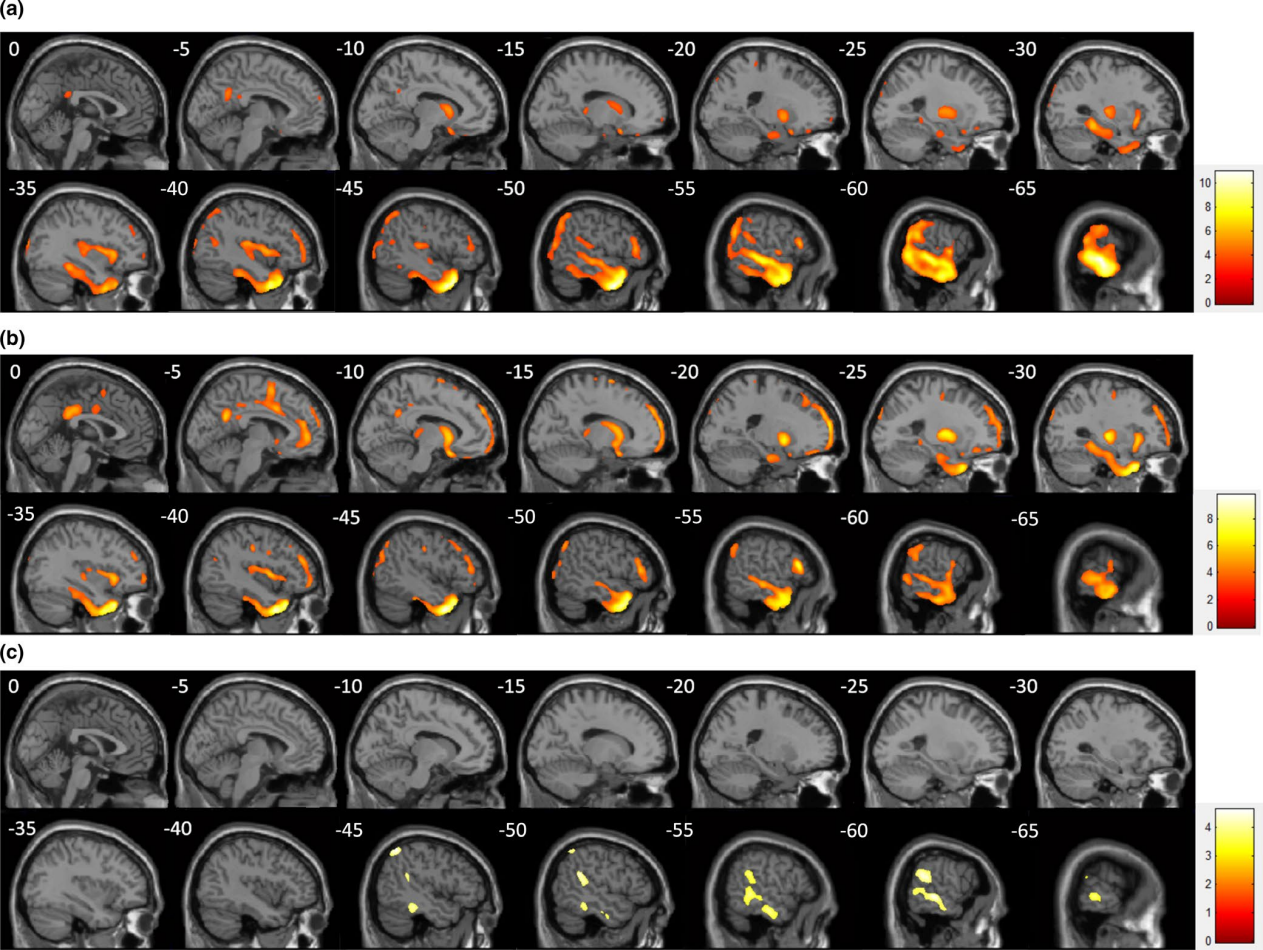
Statistical parametric mapping analysis between patients with ictal head version and controls (a), between patients without head version and controls (b), and the intergroup comparison (c). Compared with the controls, both version (+) and (−) showed extensive hypometabolism. Compared with the version (−) group, in the version (+) group, the regions with more hypometabolism included the MTN and PTN, SMG and IPL, according to the AAL atlas

Whole‐brain SPM analysis showed significantly higher hypometabolism in the 21 patients with head version than in the healthy subjects; affected regions included the ipsilateral pars triangularis, anterior and posterior insula, amygdala, parahippocampal gyrus, hippocampus, ATN, MTN and PTN, SMG, inferior parietal lobe (IPL) and bilateral putamen, and caudate based on the AAL atlas. The regions with hypometabolism were similar between the healthy controls and the 37 patients in the version (−) group except for the cingulate cortex, inferior parietal lobe, and PTN. The range of hypometabolism in the SMG was much smaller (Figure 2).

Compared with the version (−) group, in the version (+) group, the regions with more hypometabolism included the MTN and PTN, SMG, and IPL (Figure 2). The statistical threshold of these two comparisons was set to *p* < .001 and was corrected by FDR < 0.05. In the version (+) group, no significant difference was found between ipsilateral and contralateral version patients.

### 3.3 Ictal SEEG analysis

A total of 22 patients underwent SEEG electrode implantation. In the patients with ictal head version, 117 electrodes were implanted, 58 of which covered the temporal neocortex. In the version (−) group, 86 electrodes were implanted, including 50 temporal neocortex electrodes. Ictal low‐voltage fast activity (LVFA) was the collective pattern of seizure onset. In some seizures, preictal spiking, a burst of poly spikes or DC shift occurred before LVFA (Lagarde et al., 2016). The SOZ was thought to be the hippocampus in all seizures according to visual analysis. The time from SEEG onset, defined as the onset of rapid discharge, to the onset of head version was 44.81 ± 16.30 s (range: 17–106 s).

**Figure 3:**
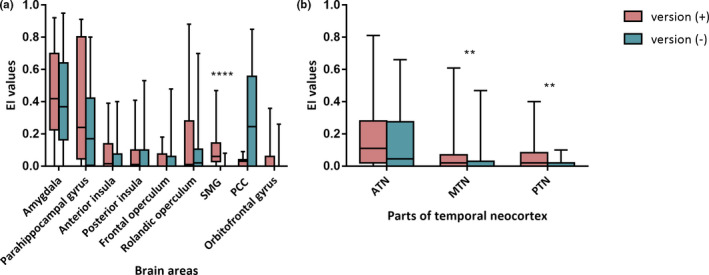
Boxplot of the EI values among seizures in each group and diverse ROIs. (a) Comparison of EI values between groups in the brain areas outside the temporal neocortex. The SMG is a unique ROI that showed a significant difference. (b) (a) Comparison of EI values between groups in the ATN, MTN, and PTN. The EI values in the MTN and PTN were higher in the version (+) group than in the version (−) group. **p < .01; ****p < .0001

Totally, 94 seizures were captured. Brain regions with EI = 1 were considered SOZ. Coincident SOZ was judged based on visual analysis and EI values in 87 seizures that were amenable to analysis, including 33 with ictal head version (version (+)) and 54 without (version (−)). EI values were calculated from several brain regions, and intergroup comparisons were made in each brain region. The numbers of seizures analyzed in version (+)/(−) were 33/52 in the amygdala, 14/23 in the parahippocampal gyrus, 28/48 in the anterior insula, 29/50 in the posterior insular, 17/29 in the frontal operculum, 19/34 in the rolandic operculum, 14/21 in the SMG, 17/17 in the PCC, 24/25 in the OFC, 63/86 in the ATN, 66/111 in the MTN, and 34/62 in the PTN. An intergroup statistical analysis of the EI values among seizures is shown in Figure 3. The EI values were significantly higher in the version (+) group than in the version (−) group in the SMG (median 0.060 vs. 0, *p* = .000), MTN (median 0.020 vs. 0, *p* = .008), and PTN (median 0.020 vs. 0, *p* = .002). No significant difference was found in other brain regions.

### 3.4 Electroclinical correlations

**Figure 4:**
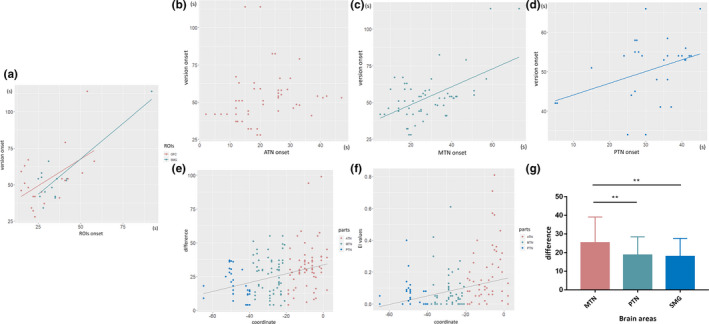
(a) A linear correlation was found between the version onset time and the onset times in the following ROIs: OFC and SMG. The relationship between onset times of version and the temporal neocortex is shown in (b–d). Correlations were observed in the MTN and PTN. A linear relationship was observed between EI (e) and the difference from version onset to temporal neocortex onset (f) and the y‐axis of the MNI coordinate. (g) Histograms of differences observed in the MTN, PTN, and SMG. The difference in the MTN was significantly higher than that observed in the PTN and SMG. **p < .01

Correlation analyses between the onset times in each ROI and head version were performed on all the ROIs mentioned above. Statistical analysis showed a linear relationship between head version onset and the ipsilateral onset time in the SMG (Pearson correlation coefficient *R* ^2^ = .747, *p* = .000) and OFC (*R* ^2^ = .275, *p* = .015) (Figure 4a). In the neocortex of the temporal lobe, the MTN (*R* ^2^ = .302, *p* = .000) and PTN (*R* ^2^ = .142, *p* = .029) also presented similar results (Figure 4c,d). In contrast, there was no relationship between the onset of head version and ROIs in other cortical areas, such as the amygdala, parahippocampal gyrus, anterior and posterior insula, frontal and rolandic operculum, PCC, or ATN.

Furthermore, to clarify the network of temporal neocortex involved during head version, we analyzed the linear relationship between EI, the difference between version onset and temporal neocortex onset, and the *y*‐axis of the MNI coordinate. There was a correlation between EI and MNI coordinates in the integral temporal neocortex (*R* ^2^ = .123, *p* = .000). This relationship showed a trend wherein smaller coordinate values (those closer to the posterior part of the neocortex) were associated with smaller EI values. Statistical analysis showed a linear relationship between this difference and MNI coordinates in the integral neocortex (*R* ^2^ = .124, *p* = .000) (Figure 4e,f). The trend was similar to that observed for EI.

The results described above suggest that the MTN, PTN, and SMG play important roles in the network of ictal head version. To clarify the pathway of discharge propagation, we compared the difference between version onset and ROI onset in three brain regions. The results showed that the difference in the MTN (25.38 ± 13.59) was larger than that in the PTN (18.71 ± 9.18) and SMG (18.04 ± 9.60) (*F* = 4.417, *p* = .014) (Figure 4g).

## 4 DISCUSSION

Ictal head version is a common symptom in epileptic seizures and is often accompanied by versive eye movements (Baysal‐Kirac et al., 2015; Marks & Laxer, 1998). Versive head rotation is present in nearly half of all patients within temporal lobe epilepsy, and among these patients, these movements occur soon (≤10 s) before focal to bilateral seizures in 27% of patients (Marks & Laxer, 1998). In our spectrum, ictal head version occurred in approximately one‐third of the patients at both the patient and seizure levels. At present, most studies on head version use semiology, scalp electroencephalogram, and imaging to clarify its localizing value (Marks & Laxer, 1998; Ochs, Gloor, Quesney, Ives, & Olivier, 1984; Remi et al., 2011; Yu et al., 2001). However, no SEEG study has elaborated on the symptomatogenic zone and the network involved in ictal head version in temporal lobe epilepsy.

Previous studies on head version in frontal lobe epilepsy have shown that it may be caused by the spread of epileptic discharges to the FEF (Godoy et al., 1990; Remi et al., 2011). Evidence obtained from electrical stimulation also suggests that stimulation of the FEF causes head version (Godoy et al., 1990; Robinson & Fuchs, 1969). However, the pattern of head version is not fixed. Both ipsilateral and contralateral versions can be induced by the stimulation, although ipsilateral version is dominant. Moreover, head version can be either induced or followed by eye version (Godoy et al., 1990; Penfield & Jasper, 1954). Hence, the FEF is considered a symptomatogenic zone of head‐eye version according to results obtained in both frontal and temporal lobe epilepsies (Remi et al., 2011).

However, the conclusions above are based on the study of frontal lobe epilepsy rather than MTLE. In our study, compared with the healthy control and version (−) groups, in the version (+) group, the FEF failed to show hypometabolism in the PET. This leads us to suspect that the FEF may not be a symptomatogenic zone of head version in MTLE. At present, it is thought that the range of PET hypometabolism reflects the spread of ictal discharges through pathways (Chassoux et al., 2004; Wang et al., 2019). Through PET analysis, we found that the range of hypometabolism observed in the version (+) group was wider than that observed in the version (−) group. The main differences were in the middle and posterior parts of the temporal neocortex and the SMG. Previous studies have suggested that a broader diversity of electrical patterns occurs in the ipsilateral version than in the contralateral version, thus prompting the involvement of the basal ganglia (Dobesberger et al., 2005; Remi et al., 2011). However, no significant metabolic divergences were found when we compared ipsilateral and contralateral versions. This may be due to the small number of cases in our study or other mechanisms, which was a potential limitation of the study. The specific mechanisms still need to be further explored.

Furthermore, to validate the results obtained in PET, we analyzed SEEG signals, which have better temporal and spatial resolution (Lagarde et al., 2018). EI algorithm determines that it plays an important role in the measurement of the epilepsy network (Bartolomei et al., 2008, 2011). In addition to the detection of the SOZ, EI was calculated to measure the propagation of the epileptic discharges (Bartolomei et al., 2011; Lagarde et al., 2018). But EI is calculated according to the energy ratio of high‐ and low‐frequency signals. If the symptom occurs in the late stage, the EEG mainly shows low‐frequency rhythms. At this time, EI has limitations to analyze the network. Electroclinical regression and functional connectivity are often needed. The EI values of several brain regions were compared between the version (+) and (−) groups. The results showed that significant differences were observed in the MTN and PTN and the SMG. This result was consistent with that obtained in the PET analysis. In addition, we analyzed the correlation between the onset time of ROIs and version. The results showed that results in the OFC, SMG, and the MTN and PTN were correlated. However, neither PET nor EI analysis found any difference in OFC between the version (+) and (−) groups. Based on these results, we conclude that the MTN, PTN, and SMG contribute to the generation of head version in MTLE.

At present, several eye regions have been confirmed in humans, including the FEF, supplementary eye field (SEF), cingulate eye field (CEF), and intraparietal sulcus (IPS) (Amiez & Petrides, 2009; Papadopoulos et al., 2018). Some of these regions are related to the control of head and eye movements (Peuskens et al., 2001; Saito et al., 1986). Apart from the FEF, the IPS attracts attention due to its function in visually guided motor control and heading estimation in monkeys and human beings (Grefkes, Weiss, Zilles, & Fink, 2002; Motter & Mountcastle, 1981; Peuskens et al., 2001). The IPS is a uniquely active region in some special movements, such as the detection of visual targets (Shulman et al., 2003). The PET results obtained in our study show that both the SMG and the IPL (the banks of the IPS in Uddin's study (Uddin et al., 2010)) based on the AAL atlas are involved in the generation of head version. We consider the IPS area (rather than the FEF) to be the symptomatogenic zone of head version in MTLE. The SMG is an important part of the network ranging from the MTL to the IPS due to its functional connectivity to the IPS (Uddin et al., 2010). This explains the high proportion of head version observed in parietal lobe epilepsy (Bartolomei et al., 2011). Moreover, this explains the hypometabolism observed in the SMG in MTLE. Using SEEG, we further confirmed the involvement of the SMG in ictal head version. However, SEEG, which was used to locate the SOZ, cannot be generally implanted into the IPS. Thus, we unfortunately could not confirm the involvement of the IPS based on SEEG.

The middle and posterior parts of the neocortex are involved in the network affected in ictal head version. These areas are close to the middle temporal area (MT) and medial superior temporal area (MST) (Huk, Dougherty, & Heeger, 2002), both of which contribute to the processing of optic flow stimuli (Dubner & Zeki, 1971; Lagae, Maes, Raiguel, Xiao, & Orban, 1994). Putative positions of the human MT and MST are typically placed on the posterior/ventral and anterior/dorsal banks of the dorsal/posterior limb of the inferior temporal sulcus (Huk et al., 2002). Studies have indicated that the MT and MST are associated with eye and head movements (Bradley, Maxwell, Andersen, Banks, & Shenoy, 1996; Britten & van Wezel, 1998). Zhang et al. argued that ipsiversive eye deviation could be an initial clinical sign of inferoposterior temporal lobe epilepsy and could be attributed to the involvement of the human MT/MST complex based on two SEEG‐analyzed cases (Zhang et al., 2017). This prompted us to consider whether the MT/MST is in the network involved in ictal head version. In an fMRI study, the MT and IPS were activated simultaneously during heading‐dimming tasks (Peuskens et al., 2001). This indicates that there are both anatomical (Maunsell & van Essen, 1983) and functional connections between the MT/MST and IPS regions. However, from the perspective of PET analysis, were found no significant difference in the MT/MST region between the version (+) and (−) groups. This suggests that the MT/MST may not be involved in the generation of ictal head version in MTLE.

To delineate the network involved in ictal head version, we chose the MNI coordinates of the temporal neocortex as independent variables and performed a regression analysis of onset time differences and EI values. The results based on both EI and onset time differences indicated that the propagation of ictal charges occurs from anterior to posterior along the temporal neocortex. The anatomical functional connectivity between the PCC and hippocampus was also confirmed (Beckmann, Johansen‐Berg, & Rushworth, 2009; Enatsu et al., 2014), and we considered the status of the PCC in the version network. Using PET and SEEG analysis, we showed that the PCC was not involved in electrical propagation from the hippocampus to the SMG. Yoo et al. also confirmed the involvement or more prominent activation of the posterior‐lateral temporal regions prior to propagation to the other cortical regions (Yoo et al., 2014). Compared with the onset differences between the MTN, PTN, and SMG, the onset difference observed in the MTN was significantly larger than that observed in the other two groups. This implies that the MTN is located upstream of the PTN and SMG in the version network.

## 5 CONCLUSION

The generation of ictal head version is attributed to the propagation of ictal discharges to the IPS area. Discharge from hippocampus to IPS must be a complex propagation network. Through the analysis of PET and SEEG signals, we propose that the network of version originates from mesial temporal lobe structures, passes through the MTN, PTN, and SMG, in turn, and likely ends in the IPS.

## CONFLICT OF INTEREST

None of the authors has any conflict of interest to disclose. We confirm that we have read the Journal's position on issues involved in ethical publication and affirm that this report is consistent with those guidelines.

## AUTHOR CONTRIBUTIONS

Yao Wang analyzed the data, acquisition of data, and drafted the manuscript for intellectual content; Xiu Wang involved in acquisition of data and statistical analysis; Lin Sang played a major role in the acquisition of data; Chao Zhang involved in acquisition of data, analysis of data, and obtaining funding; Wen‐han Hu designed the study and interpreted the data; Xiao‐qiu Shao contributed to study concept and interpretation of data; Feng Wang involved in statistical analysis and obtaining funding; Lin Ai played a major role in the acquisition of data; Jian‐guo Zhang supervised the study; Kai Zhang obtained funding, supervised the study, designed the study, and interpreted the data.

### Peer Review

The peer review history for this article is available at https://publons.com/publon/10.1002/brb3.1820.

## Data Availability

The data that support the findings of this study are available on request from the corresponding author. The data are not publicly available due to privacy or ethical restrictions.
